# Establishing a pediatric cardiac intensive care unit - Special considerations in a limited resources environment

**DOI:** 10.4103/0974-2069.64374

**Published:** 2010

**Authors:** Rakhi Balachandran, Suresh G Nair, R Krishna Kumar

**Affiliations:** Pediatric Cardiac Intensive Care, Amrita Institute of Medical Sciences, Kochi, Kerala, India; 1 Department of Cardiac Anesthesia, Amrita Institute of Medical Sciences, Kochi, Kerala, India; 2 Pediatric Cardiology, Amrita Institute of Medical Sciences, Kochi, Kerala, India

**Keywords:** Congenital heart surgery, critical care, developing country

## Abstract

Pediatric cardiac intensive care has evolved as a distinct discipline in well-established pediatric cardiac programs in developed nations. With increasing demand for pediatric heart surgery in emerging economies, a number of new programs are being established. The development of robust pediatric cardiac intensive care units (PCICU) is critical to the success of these programs. Because of substantial resource limitations existing models of PCICU care cannot be applied in their existing forms and structure. A number of challenges need to be addressed to deliver pediatric cardiac intensive care in the developing world. Limitations in infrastructure, human, and material resources call for a number of innovations and adaptations. Additionally, a variety of strategies are required to minimize costs of care to the individual patient. This review provides a framework for the establishment of a new PCICU program in face of resource limitations typically encountered in the developing world and emerging economies.

## INTRODUCTION

The care of children and adults with congenital heart disease has progressed by leaps and bounds in the last 60 years.[1] With developments in perfusion technology and refinement of surgical techniques, most complex congenital heart malformations can be repaired in the present era. Parallel developments in pediatric cardiology such as early diagnosis and rapid stabilization, improvements in imaging, advanced interventional techniques, and newer treatment options for pulmonary artery hypertension and heart failure have also complemented the growth in this field.

Since the origin of the first dedicated pediatric intensive care units in 1950s, the field of pediatric intensive care has been expanding and sprouting new subspecialties.[2,3] The concept of dedicated pediatric cardiac intensive care units (PCICU) originated from the unique requirements for management of children after cardiac surgery. In the early years of development of congenital heart surgery, pediatric cardiac surgeons have been primarily responsible for postoperative intensive care. Over the past three decades, other pediatric cardiac professionals (cardiology, cardiac anesthesia, critical care nursing, respiratory therapy, and others) have started to contribute increasingly to the care of these patients. Thus, pediatric cardiac intensive care emerged as a new subspecialty to cater to the unique needs of children with congenital and acquired heart disease.[4]

## MODELS OF PEDIATRIC CARDIAC INTENSIVE CARE

The most common model of pediatric cardiac critical care, especially in developed nations, is through a specialized pediatric cardiac intensive care facility located in a children's hospital. The services are provided by a multidisciplinary team that includes pediatric cardiologists, pediatric cardiac surgeons, intensivists, critical care nurses, respiratory therapists, and other support personnel.[2] The second model is where the pediatric cardiac critical care is a part of a general pediatric intensive care unit.[5] These models are successfully used to deliver pediatric cardiac intensive care in several well established and large pediatric heart programs in United States, Canada, Europe, and Australia. Many of these units run focused pediatric cardiac intensive care training programs.[5]

In developing nations with limited infrastructure, human, and material resources, pediatric cardiac intensive care is yet to take roots as a distinctive discipline. As a result, many models exist. Pediatric heart programs are often attached to well-established adult cardiology and cardiac surgery programs, and PCICU care is sometimes delivered in a common setting with shared space, infrastructure, and personnel. In small private establishments, it is delivered by a small group of professionals (mostly anesthesiologists) attached to the surgical unit. International collaboration with established programs in the West is often used by many evolving pediatric cardiac programs in developing nations to organize PCICU.[6,7] These centers specially focus on establishment of systems, protocols, and local staff development.

## WHY IS THERE A NEED FOR A DEDICATED PEDIATRIC CARDIAC INTENSIVE CARE UNIT?

1. With facilities for accurate diagnosis and scope of complete correction, more and more children are undergoing surgical treatment for congenital heart diseases, so there is increasing demand for dedicated personnel for the specialized intensive care of these critically ill children.[5]
2. A dedicated team dictating specialized intensive care in a defined unit has translated into better outcomes in several centers.[1,8-10] The team-oriented focus allows all members of the unit to have intellectual ownership in the heart program and contribute to the care of the child, thus exploiting the full advantage of each discipline.
3. The trend toward early primary surgical repair (during early infancy or newborn period) of many congenital heart lesions is now well established.[11] In several centers, where pediatric cardiac care is linked to busy adult surgical programs, health-care professionals trained in adult cardiac care may not be able to provide optimal care for neonates and small infants. Hence, it becomes imperative to specially train a group of physicians, nurses, and supportive staff who can handle this very young population.

## REQUIREMENTS FOR AN IDEAL PCICU AND IMPROVIZATIONS IN THE FACE OF RESOURCE LIMITATIONS

### Unit design

The PCICU should be geographically distinct and designed to receive children with congenital and acquired heart disease. The bed strength should be decided on the basis of the surgical case volumes. A 6-10 bed ICU with scope for future expansion is a reasonable start. Proximity to the operating rooms and cardiac catheterization laboratory are desirable for obvious reasons. Easy accessibility to the elevators is mandatory to facilitate transport to and from other areas of the hospital. The access to the PCICU should be monitored to maintain patient and staff safety and confidentiality.[12]

The on-call physician's room, staff rest rooms, and family waiting areas should also be close to the ICU. The design should also allow provision for at least two isolation rooms, clean and dirty utility rooms, nourishment preparation areas, medication station, narcotic locker, and a refrigerator. A conference room for staff education, conferences, and case discussions is desirable.

### Central station

The central station should offer good visibility to all patient beds and overhead monitors. Adequate space should be allowed for the doctors and nurses to write case details and order sheets. It should have a central hemodynamic display monitor. At least two computers should be available for electronic data entry, clerical works, and communication. Two telephone lines and intercom facilities should be available.

### Bedside facilities

Beds should be arranged in a manner which allows visibility from a central care station. Patient area in the open PCICU should be 150-200 sq ft.[13] There should be adequate space around the bed for accommodating ventilators, syringe pumps, and IV poles. There should be easy accessibility to the head end of the bed/crib for emergency airway management. Bassinets with overhead radiant warmers are necessary for receiving neonates and small infants. There should be enough space for performing routine ICU procedures (e.g. central line placement, intercostal drain insertion, etc). Monitoring equipment can be wall mounted to save physical space, to avoid overcrowding, and to allow easy visibility from a distant care station. Sufficient washbasins should be available to allow frequent hand washing.

Electrical power from an uninterrupted source (UPS), oxygen, medical compressed air and vacuum outlets sufficient in number to supply all necessary equipment should meet regulatory standards.[12] Backup power supply and medical gas supply should be immediately available. The unit should be centrally air-conditioned.

### Equipments

A list of basic equipments and utilities to support the needs of the pediatric cardiac patients under normal and emergency conditions is provided in Table 1.[12,13] In addition, cardiac intensive care units also need specialized equipments like echocardiography machines with trans-esophageal probes, nitric oxide cylinders, and nitric oxide delivery systems.[14] Although ideally, paraphernalia for institution of mechanical circulatory support is desirable, there are specific concerns in developing resource intensive extracorporeal membrane oxygenator (ECMO) services in many centers in the developing world. There are several programs that have achieved excellent outcomes without the availability of ECMO. It is perhaps reasonable to state that ECMO services should only be considered once a certain basic standard of care is achieved with congenital heart surgery.

**Table 1:** Essential equipment for the pediatric cardiac intensive care unit

Monitoring equipment	
Multi-channel patient monitors (capable of continuous measurement of ECG, HR, CVP, ABP, SPO_2_, ETCO_2_, temperature, RR, should have facility for setting appropriate alarm limits, audible alarms, should have memory and display trends, print out feature should be available)	
NIBP cuffs and hoses
SPO_2_ sensors-pediatric
Temperature probes
ECG leads and ECG cables
Transport monitors	

Procedural equipments	Respiratory equipment

Intravascular catheters	Ventilators with waveform displays
Central venous catheters-	Non-invasive ventilators
4 Fr, 5.5 Fr, 7 Fr	Breathing circuits
Transducer assembly	Ambu bags
Pressure extension lines	Suction catheters
Disposable guide wires	Laryngoscopes
Dressing tray	Endotracheal tubes
Suture removal set	Stylet
Central line tray	Bougie
Emergency sternotomy set	Suction catheters
Peritoneal dialysis catheters	Humidifiers
ICD tray	Nebulizer kit
IV infusion sets	Oxygen masks
Blood transfusion sets	Nasal cannulae
Three way stopcocks	Bi-level non-invasive ventilation
Portable lights	Oxygen cylinders
	Vibrator
	Spirometers
	Continuous oxygen analyzers and alarms
	Tracheostomy set and tracheostomy tubes
	Flexible fiberoptic bronchoscope

Specialized equipments for cardiac ICU	Portable equipment

Pacemaker: Single chamber and dual chamber	Crash cart Defibrillator and cardioverter
Pacing cables	Beds and bassinets
Transvenous pacing set	Syringe pumps
Cardiac output monitor	IV poles with wheels
Internal paddles for defibrillator	Suction machine Infant warmers
Nitric oxide cylinders and delivery system	Overhead warmers Warming blankets and Bair hugger.
Echo cardiography machine	Infant weighing machine
ECG machine	Oxygen cylinders
USG machine-optional	Bedside locker
Hand held Doppler	Bedside table
	Bedside chair
	Clocks and calendars

### Administrative structure and work pattern in the PCICU

The PCICU should have a well-defined administrative structure and staff pattern of its own. There are no published recommendations, but most of available evidence encourages developing a unit with a physician/intensivist as the leader/coordinator of the intensive care team.[1,8,9,12]

### Leadership-(Intensivist in-charge) of the PCICU

In a developing nation, a specially trained pediatric cardiac intensivist is a rarity. Under this circumstance, the leadership for the PCICU should preferably come from a physician trained in one of the pediatric cardiac subspecialties. Recognizing the paucity of dedicated cardiac surgeons and intensity of their involvement for prolonged periods in the operation room, it is unrealistic to expect the surgeon to take up this role. A pragmatic approach is to have a cardiac anesthesiologist or pediatric cardiologist specially focus on intensive care and, over some time, develop as a leader of the PCICU.

The intensivist in-charge can be expected to shoulder the following responsibilities.[12,13]

1. Act as a multidisciplinary team leader, coordinating care provided by the members of the team.
2. Establishing policies and protocols in collaboration with members of other subspecialties in the team.
3. Promote the implementation of the policies and protocols including admission and discharge criteria.
4. Provide primary or consultative care for all the PCICU patients along with the physician on call in the ICU.
5. Participate in quality improvement activities.
6. Co-ordinate and participate in staff education and research.
7. Maintain a database that describes unit experience and performance.
8. Supervise cost containment measures in the intensive care delivery.
9. Plan staffing requirements and work to ensure short-term and long-term adequacy of nursing and physician staff.

### Other physician staff

An in-house physician (resident or fellow in pediatric cardiology or anesthesiology) skilled to provide emergency care to critically ill children with congenital heart disease should provide continuous cover. He or she should not have any other simultaneous responsibilities while on call in the intensive care unit.[13]

### Nursing staff

The PCICU team should have a cadre of specially trained nurses of its own led by a senior nurse (Nurse in Charge) expertise in managing critically ill children with congenital heart disease. The work pattern should be organized as an 8 or 12 h shift based on the availability of staff nurses in the critical care team. There should preferably be a nurse leader for every shift (team leader). The ratio of nurses to patients is typically 1:1 or 1:2 depending on patient acuity and the number of staff available per shift.[15]

The *nurse-in-charge* should be responsible for the nursing program, delegation of roles, and responsibilities to members of the nursing team, implementation of policies and procedures, quality assurance, the provision of supplies and equipment, and staff education and training. The *bed-side* nurse should be entrusted with the care-giving function. He or she should perform repeated clinical assessment of the patient, collect vital information, communicate relevant information to the physician staff and other members of the team, and provide compassionate care to the critically ill child. PCICU nurses should also interact with the family members and participate in patient and family education.

Ideally, the pediatric cardiac critical care nurse should possess basic knowledge about the anatomy and pathophysiology of the common congenital heart diseases, the surgical treatment for the disease, cardiopulmonary bypass and its effects on organ systems, hemodynamic monitoring of cardiac patients, essentials of pace makers and cardiac pacing, pharmacology of cardiovascular drugs, recognition and management of the common post operative complications, mechanical support of the heart, diagnosis and management of cardiac arrhythmias, and cardiopulmonary resuscitation techniques. However, in real life situations, this level of expertise is acquired over a considerable period of time. The value of experience is considerable and needs to be recognized and compensated adequately by the administration.

### Other ancillary staff

All PCICUs should be regularly supported by respiratory therapists, nutritionists, physiotherapists, medical social workers, and nursing assistants for comprehensive patient care.[16] Radiographers, ECG technicians, biomedical engineers, and electrical engineers should be easily accessible to meet emergencies/problems in the respective areas. A unit secretary should be appointed to carry out communication, electronic data entry, as well as paperwork involved in the smooth functioning of the unit.

### Day-to-day care in the PCICU

Once the necessary infrastructure and the manpower are in place, a system for the smooth functioning of the unit should be evolved. While initiating a new program on a collaborative approach of various subspecialties, it would be logical to evolve a daily work pattern by discussion with senior members of each of the subspecialties of the team. There should be a forum where all the members of the care team can assemble together, present relevant clinical information, combine individual clinical expertise, conduct healthy discussions, and formulate a care plan for each patient in the PCICU. This, in the critical care setting is best achieved at the bedside by conducting multidisciplinary rounds at the bedside.[16,17]

A proposed suggestion for the work pattern is given below.

- The daily care team
- The intensivist in charge
- Physician on call
- Pediatric cardiac surgeon/surgical fellow
- Pediatric cardiologist
- Nurse in charge
- Bedside nurse
- Respiratory therapist
- Nutrition specialist
- Medical social worker

### Pre-rounding phase

The day starts with the nursing shift change over during which the night duty nurse hands over the patient details to the day duty staff. There should be minimal interruption of the nurses by physicians or other staff members to avoid communication errors. The morning blood draws should be completed early so that reports are ready for analysis during rounding. The physician staff collects information about the current status of the patient by communication with the night duty person, clinical assessment, review of flow sheets and lab reports, issue any physician orders if required. After this, the primary care giver should synthesize data in a daily progress note and formulate a tentative plan for the day.

### Multidisciplinary rounds

The PCICU intensivist should lead the rounds. Interruptions during rounds should be avoided (except emergencies). The physician on call makes a comprehensive presentation of the patient data to the team in a uniformly structured format that summarizes the diagnosis, operation performed, and most significant events till date. The past 24 h is dealt with in greater detail and a complete multi-system assessment of the patient's current status is made. With practice, this presentation can be accomplished quite quickly. Once the data are presented, a group assessment of the child in the disease process (acute, plateau, recovery phase) should be made and the child's greatest safety risk should be identified. Along with this, invasive catheters and drainage tubes, adequacy of pain control, sedation, ventilation, inotropic supports, current medications, and transfer or discharge needs should be reviewed. Based on the inputs of all the members of the team, the intensivist should formulate a care plan for the day. The care plan along with the daily medication orders should be written down by a member of the team and read back to the team for better clarity and to minimize ambiguity in decision making. Incorporating a daily “goal chart” will help to keep the care team in focus.[18]

The daily team should work toward achieving the set goals. Variations in care plan that may arise due to alterations in the patient's status during the course of the day should be communicated to the members of the team as appropriate. In situations where the physician on call works on 12 h shifts, evening “sign-out” rounds would be appropriate to exchange clinical information and to identify short-term goals for the night.

### Communication with the family

Family centered care is becoming popular in the pediatric intensive care setting.[19] Several well-established centers are allowing the presence of family members during the decision-making rounds. There are no definite policies in many hospitals to address this issue. However, it would be ideal if the caregivers keep the family updated regarding the progress of the patient on a daily basis at the very least. The current physical status of the patient, details of illness, major concerns in the next 24 h, details of the care plan, and the anticipated duration of ICU stay should be elaborated as appropriate. Every single intervention, diagnostic test, therapeutic measure, or additional stay in the ICU adds to the economic burden to the family. In countries with limited resources, the economic implications of the decisions taken in the care plan should also be clarified with the family.

### Establishing policies and procedure

Controlling practice variation is a key variable in delivering quality patient care.[18] Basic policies and protocols should be evolved through discussions with senior members of the team, and should be used to streamline the repetitive procedures done in the ICU (e.g. potassium correction, care of arterial line, pain management). These policies and protocols can also serve as an educational tool to the junior members and trainees of the ICU team and should be periodically reviewed and updated according to the currently available evidence. Notwithstanding guidelines and protocols, one should have an open mind to critically evaluate each situation and provide an individualized approach to patient care.[16]

### Data collection system

There should be a collective effort from all members of the team to maintain accurate records. There should be complete and clear recording of the patient status and procedures by the primary caregivers (physician and nursing staff). Electronic medical records system is becoming increasingly popular even in developing nations to minimize recording errors, to store a large data, and allow easy retrieval for periodic review of performance.[17,18,20]

### Quality control

Continuous quality improvement is a proactive process whereby all structures, processes and clinical activities occurring in critical care setting should be periodically evaluated.[21] The best variables for measuring quality are still unclear. However, conventional indicators of outcome after congenital heart surgery largely acceptable to most centers should be tracked. Readmission rates to ICU within 24 h of transfer during a single hospital stay, re-intubation rate, duration of mechanical ventilation, length of stay in ICU, frequency of catheter related blood stream infections are some of the variables that can be used as a measure of quality improvement.[18,22] Regular morbidity-mortality meetings allow performance analysis and help define scope for improvement.[16,23] Errors and adverse events occurring during critically ill patient care have been recognized as major concerns in intensive care.[22] These areas should be considered as targets for quality improvement in the ICU.

### Staff education

The initiation of staff development and training should begin much before the actual implementation of the program. It would be ideal for a senior member of each of the subspecialties to visit a well-established centre and observe the day-to-day functioning of the unit. Arranging a short period of observation or overseas training can help substantially when a new program is being established with limited resources. After establishment, continuing an 'in-house' education programs is vital for all the medical and paramedical personnel in the unit. This can be accomplished in several ways. The intensivist-in-charge can co-ordinate the staff training program. All the senior members of the various subspecialties have to be involved in academic discussions and formal lecture sessions. The junior trainees of the team including the nursing staff can be encouraged to present clinical cases and participate in care planning and implementation. Special knowledge areas like single ventricle physiology, pulmonary hypertension, mechanical support, arrhythmia management, cardiopulmonary resuscitation, pacemaker use, etc. should be reviewed periodically to keep the care takers better oriented and prepared.[14] The senior staff should encourage and guide trainees and nursing staff to do research projects. All members of the program should be encouraged to attend regional or national conferences with course content pertinent to the PCICU care regularly.

## SPECIAL CHALLENGES

### Role of leadership in the PCICU

When designing the organizational structure of the ICU, the administrative hierarchy should be clearly delineated. The role of a leader is vital to streamline the multidisciplinary teamwork. The leader should be a well-accepted member of the team who has the confidence of other specialties and their consultants. The leader should also possess skills in management, organization, and mentorship.[16,24,25] The role of leader should incorporate the task behavior of delegating roles and responsibilities to the members of the team, and a relationship behavior through which team is held together.[24] In the initial phase of the unit, the leader should work along with the team participating directly in the task responsibilities. As the team becomes competent and mature he or she can switch start delegating leadership. However, in emergencies and crises (resuscitation in cardiac arrest), the leader should ideally be available to guide the team.

### Ensuring team harmony and managing conflicts in the unit

One of the requisites, particularly in the formative period of the PCICU is to establish a healthy working relationship among the team members who vary in their professional profile, clinical skills, knowledge, and managerial capacities. Conflicts and controversies are likely to arise when individuals with diverse ideas and diverse profiles work together. There is no rule of thumb to avoid such situations. However, this can be minimized through conscious effort. First of all, it has to be instilled into the team members that they are working toward the common goal of the well-being of the patient. The role of each member in striving for this goal has to be clearly delineated. Secondly, standard operating procedures (e.g. policies and protocols) have to be developed to minimize diversities in practice. Thirdly, concise and clear communication should be practiced between various members of the team at all levels.[24] The role of a leader in ensuring effective communication in the unit is very vital to win the trust of the team members and encourage close co-operation and teamwork.

### Dealing with complications

Children undergoing congenital heart surgery are vulnerable to many complications and adverse events. These may be brought about by chance, due to the specific nature of the underlying illness, technical failures, or human errors.[22] When such an event occurs, attempts to blame individuals involved in the care pathway can generate unnecessary conflicts, upset the group morale, and add to the work stress of the team members. It would be ideal and in the best interest of the team to address these events through constructive criticism. The event or complication should be identified, root cause analysis should be performed, and changes be made in the protocols and systems to prevent similar events in the future. For example, in the event of a accidental hyperkalemic cardiac arrest, re-evaluating potassium replacement guidelines, evolving policies for monitoring and administration of potassium, emphasizing the importance to check for related variables like urine output, medications causing hyperkalemia, and educating the team to handle the management of hyperkalemia are improvement strategies which can be adopted.

### Decision making in the ICU

Clinical decision making for a critical ill child requires physicians of various subspecialties to combine their clinical skills, past experiences, and knowledge to analyze the situation at hand, to decide the best course of action compatible with current practice guidelines, and translate them into an action plan at the bedside.[20] The major decisions in the course of care delivery in a PCICU evolve after multidisciplinary rounds. These decisions should be clearly documented and communicated to the primary care givers who should execute the plan. However, in acute care setting where changes in patient status from the expected course can occur at any time, the physician on call should be authorized to make necessary alteration in the care plan to suit the situation. The changes and the reasons for change should be promptly communicated to the responsible senior members of the team. Decisions in relation to routine events and procedures (e.g., potassium replacement, changing an invasive line, administering a sedative) can be taken as and when required. In emergency situations, the primary care giver should have the full authority to take the best course of action that he or she feels appropriate for the situation. (e.g. cardioversion for a tachyarrhythmia, changing a blocked endotracheal tube).

In a limited resource set up with manpower shortage, when the role of primary care taker falls on the junior members and trainees, there may be an inability to implement appropriate decisions in a timely fashion due to the lack of experience and the steep learning curve involved. Allowing some degree of autonomy in clinical decision making to junior team members in appropriate areas can encourage creative thinking and motivate learning process.[25] However, senior members of the team should keep a cautious watch to compensate for the learning curve in the initial phase. A good decision that has yielded a better outcome should be appreciated by the senior members of the team. This will improve motivation, commitment to patient care, and job satisfaction in the younger members of the care team.

### Retention of personnel

The field of pediatric cardiac care is consumed by an overwhelmingly growing need for more trained personnel at all levels of care in the ICU. This is especially relevant in the context of emerging nations where structured training programs and well-established intensive care units are lacking.[26] There are very few trained pediatric cardiac intensivists. There is also a considerable attrition from already existing cadre due to migration to developed nations in search of better remuneration.[27] One of the potential ways of addressing this manpower shortage is to utilize personnel from allied specialties of pediatric cardiology, cardiac anesthesiology, and neonatology to share the responsibilities in delivering pediatric cardiac critical care.[28] Structured and accredited training programs have to be developed in upcoming centers with the goal of training more health care professionals. Cardiac intensivists already working in the field should encourage and inspire interested trainees or fellows to fill existing training positions.[28] Arranging overseas training programs for interested physicians and nurses from emerging nations in collaboration with charitable organizations can be an additional motivation for learning and participation.

Most of the physicians, nurses, and support staff working in cardiac programs in developing nations are underpaid for the amount of time and effort that they invest in executing the job. In a developing country, recognizing the monetary needs and offering better incentives can help to retain currently existing personnel.

The profession of pediatric cardiac intensive care is highly demanding in terms of efficiency, skills, and long working hours. Extremes of stress and emotional exhaustion can lead to premature burn outs, especially in the acute care setting of the PCICU.[5] Efforts to prevent such burnouts with career diversification in other areas of interest (e.g. research, administration) or allow significant time away from the intensive care unit setting on a periodic basis should also be encouraged.

### Cost containment

While establishing a new unit in a developing country, the issue of effective resource utilization and cost containment assumes a overwhelming importance. In most developing nations, the cost for the ICU care constitutes one-tenth to one-sixth of the total costs of congenital heart surgery.[29] The goal of a successful intensive care team should be to reduce expenditures without compromising quality. Unless a conscious attempt is made toward this end, patient bills frequently get out of control and the program will acquire the reputation of being very expensive with the attendant risk of premature collapse. Cost containment strategies should be instituted at all stages of care. Some of the cost containment measures specific to the intensive care unit needing special attention are discussed below.

### Early extubation

Fast tracking and early extubation are gaining popularity in children undergoing congenital heart repairs.[30] This obviously minimizes duration of ventilator support with considerably reduced resource use. Early extubation strategy can be adopted in uncomplicated cases by judicious selection of anesthetic agents without compromising safety.[29] To some extent, early extubation is dependent on surgical strategies and surgical support times (bypass and cross-clamp times). For example, delayed sternal closure will typically add an additional day or two to the duration of mechanical ventilation.

### Reduction of laboratory tests

There are no definite guidelines regarding the optimal frequency of postoperative laboratory tests or arterial blood gas estimations in the PCICU. However, the unit should evolve a policy suited to the patient profile. The tests should be done more frequently in the initial critical phase to ensure patient safety and should be scaled down when the child has been significantly de-intensified [Tables 2 and 3.] Alternative strategies such as continuous ETCO_2_ monitoring, measuring blood sugar with glucostrips instead of laboratory blood testing should be tried whenever feasible. A conscious effort to avoid all unnecessary laboratory tests and chest X-rays can go a long way toward significantly reducing ICU costs. This is particularly important in situations associated with prolonged postoperative recovery.

**Table 2:** A proposed strategy for frequency of arterial blood gas sampling in the PCICU

ABG timing in the post op course	Frequency of sampling
At admission/receiving from operation room	Immediate
After institution of initial ventilator change based on first ABG	Within 30 min
During the course of mechanical ventilation	Every 4 h
Prior to extubation	Obtain sample on pressure support/ volume support mode of ventilation
Post extubation	Within 30 min
In all extubated patients	6-8 hourly
Re-intubation	Immediate
Re admission into the ICU	Immediate
In case of hypo/hyperkalemia	1-2 hrly based on severity of the electrolyte imbalance and hemodynamic disturbances.

ABG: Arterial blood gas

**Table 3:** A proposed strategy for laboratory tests in the post operative period for typical open heart operations*

Post operative days/special situations	Suggested tests	Frequency
On admission into ICU (day of surgery)	CBC, RFT, LFT, PT, APTT, INR, serum electrolytes, ECG	Immediate
	Chest X ray	Immediate and after 6 hrs
Post operative day (POD) 1	CBC, RFT	Once daily
	Chest X-ray	Twice daily
POD 2 onward	CBC	Once daily
Patients on anti-coagulants	PT, APTT, INR	Daily

CBC: Complete blood counts, APTT: Activated partial thromboplastin time, ECG: electrocardiogram, INR: International Normalized Ratio, LFT: Liver function tests, RFT: Renal function tests.

* This can be modified to suit individual situations (for example, operation for closure of atrial septal defects may require fewer tests).

### Medication management

The management of a critically ill cardiac patient requires an armamentarium of drugs including inotropes, antibiotics, anti-arrhythmic agents, vasodilators, opioids and sedative medications, and expensive gases such as nitric oxide [Table 4]. The choice of drugs and duration of treatment should be tailored to patient needs. Antibiotics and inotropes are especially likely to be overused in the ICU unless there are guidelines. Limiting the duration of peri-operative antibiotic prophylaxis to 24-48 h and carefully choosing antibiotics based on hospital antibiogram can help to minimize irrational use of antibiotics and prevent development of multi-drug resistant microorganisms.[27,29] Inotropes should be selected to suit postoperative physiology and tapered off when they are no longer required. The introduction of inhaled nitric oxide (iNO) has added a new dimension to the treatment of pulmonary hypertension associated with congenital heart disease. However, the expenses involved are quite formidable. Efforts should be made to restrict the duration of use in “indispensable situations” such as weaning off CPB or managing pulmonary artery hypertensive crises in the postoperative period. While sildenafil may potentially substitute for iNO, its use in the early postoperative period needs to be studied and standardized.

**Table 4:** Essential drugs in pediatric cardiac ICU

Resuscitation drugs	Inotropes	Vasodilators
Adrenaline	Dopamine	Sodium nitroprusside
Atropine	Dobutamine	Nitroglycerine
Calcium chloride	Adrenaline	Captopril
Calcium gluconate	Norepinephrine	Diuretics
Lignocaine	Isoprenaline	Frusemide
Sodium bicarbonate	Milrinone	Spironolactone
	Digoxin	Acetazolamide
		Metolazone
Anti-arrhythmics	Drugs affecting coagulation	Sedatives and analgesics
Adenosine	Heparin	Fentanyl
Amiodarone	Aspirin	Morphine
Bretylium	Warfarin	Midazolam
Lignocaine	Streptokinase	Paracetamol
Verapamil	Tranexamic acid	Ketamine
Magnesium sulfate	Protamine	Propofol
Respiratory medicines	Miscellaneous	Vecuronium
Salbutamol respirator solution Racemic epinephrine Budesonide respirator suspension	Aminophylline Dexamethasone, hydrocortisone Naloxone Phenytoin Phenobarbitone Indomethacin Prostaglandin	Pancuronium

### Regulating blood product usage

Unwarranted use of blood products can add to costs and increase transfusion-related complications.[31] Ultra filtration during CPB and use of cell savers can reduce transfusions and cut down cost. In the face of increased post-operative bleeding, “point of care” tests like thromboelastography should be used if available to accurately assess the coagulation defect. This will promote the judicious use of blood products and minimize unnecessary transfusions. This is yet another area where the surgeon can consciously help to reduce costs.

*Re-use of disposables and developing products locally* Imported disposables from developed nations are being widely used in open heart surgery. With increasing demands, the costs to most programs are substantial. It is important to develop indigenous technology manufacture these items locally at cheaper prices. Re-use of selected disposables like cannulae for cardiopulmonary bypass, catheters, and guide wires after appropriate sterilization is also an alternative option in a low cost environment.[27,32]

### Early de-intensification in the ICU

Once the patients are extubated and are hemodynamically stable without inotropic supports, invasive lines should be removed. Early removal of invasive lines can minimize the risk of catheter-related blood stream infections and subsequent morbidity.[33] Early enteral feeding and ambulation can facilitate early transfer from the ICU and reduce ICU charges.

### Reducing ICU stay

Sepsis, neurological complications, lung issues, pulmonary hypertension, diaphragm palsy, renal failure, and residual lesions can prolong ICU stay.[27,34] All precautions should be taken to prevent or minimize these complications in the peri-operative period. Infection control policies should be aggressively implemented to reduce peri-operative sepsis and minimize expensive antibiotic usage and thus duration of stay.[34] Early de-intensification at all levels of post-operative care ultimately aims at reduction in the total number of days spent in the ICU.

### Critical pathways

Recently, application of critical care pathways to post-operative management of children with congenital heart disease have been found to reduce resource utilization and costs of postoperative care.[30] This encompasses principles of early extubation, adequate pain control, early ambulationz and alimentation reduced ICU stays, discharge planning and teaching. Critical pathways can also help to minimize inadvertent variation in patient care in the long run.

## CONCLUSIONS

Pediatric cardiac intensive care in developing nations is still in infancy. The establishment of a pediatric cardiac intensive care unit in a limited resource environment is a major challenge. The delivery of optimal care in the backdrop of limited resources and lack of trained personnel require good teamwork, perseverance, and dedication. While awaiting the development of well-established quality institutions with robust training programs, emerging programs in developing nations should be encouraged and supported for the development of pediatric cardiac intensive care as a distinct discipline.
